# The Intricacies of Children’s Physical Activity

**DOI:** 10.1515/hukin-2015-0082

**Published:** 2015-10-14

**Authors:** Timothy A Brusseau

**Affiliations:** 1 Department of Exercise and Sport Science, Sport Pedagogy and Physical Activity Assessment Laboratory, University of Utah.

**Keywords:** physical education, pedometer, recess, pediatric, school

## Abstract

Understanding the physical activity patterns of youth is an essential step in preparing programming and interventions needed to change behavior. To date, little is known about the intricacies of youth physical activity across various physical activity segments (i.e. in school, out of school, recess, classroom physical activity, physical education, weekends, etc.). Therefore, the purpose of the study was to examine the physical activity patterns of elementary school children across various segments and during two seasons. A total of 287 fourth and fifth graders from the Southwest US wore the Yamax Digiwalker SW-200 pedometer for 7 consecutive days during the Fall and Spring seasons. Children were prompted to record their step counts when arriving and leaving school, before and after physical education and recess, as well as on the weekends. Means and standard deviations were calculated and ANOVAs and t tests were utilized to examine difference by sex, season, and segment. Youth were more active outside of school and on weekdays (p<0.05). Boys were generally more active than girls and all youth were more active during the milder Spring season. There is a clear need for Comprehensive School Physical Activity Programming and weekend physical activity opportunities. Furthermore, greater emphasis is needed on PE and across other activity segments for girls to increase their physical activity levels.

## Introduction

With increases in health risk of young people, it is essential to investigate factors such as physical activity that may contribute to these public health issues (Kriska et al., 2013). Understanding the physical activity patterns of youth is also an essential step in preparing programming and interventions needed to change behavior. Recent studies have found decreasing levels of physical activity and increases in sedentary behavior before adolescence (Basterfield et al., 2011). Researchers have also noted that physical activity peaks at the age of 12 (Tudor-Locke et al., 2011).

To combat these decreases in activity, numerous organizations have recommended the school as an ideal locale for improving physical activity behavior (CDC, 2011; NASPE, 2008). Recently, the CDC and SHAPE America have championed Comprehensive School Physical Activity Programming (AAHPERD, 2013; CDC, 2013). To date, however, very few studies have explored the intricacies of youth physical activity, especially with regard to various school activity segments (in and out of school, physical education, recess, physical education days, etc.). Currently, there have been only three studies exploring the step counts of youth across various activity segments (Brusseau et al., 2011; Brusseau et al., 2013; Tudor-Locke et al., 2006). These studies were limited to 5–6 days of weekday data during one season and only one study included weekend days (Brusseau et al., 2013).

Numerous researchers have suggested that it is important to understand the role that seasons play on youth PA as temperature and weather patterns can influence PA levels (Beighle et al., 2008; Brusseau et al., 2012; Carson et al., 2010). Furthermore, it has been suggested that a better understanding of seasonal differences may help prioritize intervention programs or school resources to meet the needs of the low active students (Beighle et al., 2012). Previous examinations of seasonal impact on youth PA have primarily found that children are more active when the weather is warmer and allows for outdoor activity and have mostly examined total daily physical activity (Beighle et al., 2008; Belanger et al., 2009; Kristensen et al., 2007; Rowlands and Hughes, 2006).

Therefore, the purpose of this manuscript was to explore the physical activity differences of youth across various physical activity segments during both the Fall and Spring seasons.

## Material and Methods

### Participants

Participants were from two elementary schools in the Southwestern US. A total of 287 (168 females) youth in grades 4 and 5 participated in the study. The youth were 9.48 ± 0.66 years old with an average BMI of 18.91 ± 4.08. Youth were 50% Caucasian and 50% ethnic minority (22% Hispanic, 15% African American, 8% American Indian, and 5% Asian/Pacific Islander). A total of 64% of youth were healthy weight, 16% overweight and 20% obese according the CDC growth charts (Ogden et al., 2002). Participants provided written assent and their parent/guardian provided informed consent. All procedures were approved by the University IRB as well as the district research board and school principals.

### Measures

The Yamax Digiwalker SW-200 Pedometer (Yamaxx, Tokyo, Japan) was used to measure student physical activity. This instrument has been shown to provide valid and reliable data in a pediatric population (Hart et al., 2011). A digital scale (Seca 882 Digital BMI Scale) and stadiometer (Seca 214 Portable Stadiometer) were used to measure weight to the nearest 0.1 kg and body height to the nearest 0.5 cm. The BMI was calculated using the formula kg/m^2^. Prior to data collection both the pedometers and scale were calibrated to ensure accuracy.

### Procedures

Prior to data collection in both the Fall and Spring, although previous research has suggested reactivity is not an issue (Prewitt et al., 2013), all students were provided a pedometer during a physical education class to become familiarized with the monitors. Participants also completed step tests (Vincent and Sidman, 2003) to ensure that the placement over the right knee accurately measured their physical activity. Data collection procedures were adapted from previous research (Tudor-Locke et al., 2006). At the beginning of the first school day of the study, youth received a pedometer with their participant ID on the monitor. Participants wore the pedometer for 7 consecutive days in the Fall and Spring seasons. They were instructed to wear their pedometer during all waking hours except when they would be doing water-based activities. At the beginning and end of each school day youth were prompted by the research team to record their step counts as well as before and after physical education classes and recess. Students then wore the pedometer at home and wore them back to school the following day. Upon recording their steps at the beginning of each school day, the youth were instructed to reset the pedometer to zero. The morning record provided the research team with a daily or 24 hour step total while the end of school record provided the researchers with an in-school total. Outside of school physical activity was computed by subtracting the in school total from the total day step counts. Recess and physical education totals were obtained by subtracting the before segment step value from the post segment value. On weekends, youth were sent home with a recording sheet that reminded youth to record their step counts each morning. Researchers made note of which days the participants had physical education classes. Data were collected in October and April representing both the Fall and Spring seasons. Recess included both 20 min after-lunch recess and afternoon 15 min recess. A physical education class was 30 minutes in length twice per week and included traditional team games and fitness activities.

### Statistical Analysis

All 287 students had at least 2 days of data collected and therefore were included in the analyses. Means and standard deviations were calculated for each season by in school, out of school, week day, weekend day, recess, physical education, and physical education day. ANOVAs and *t* tests were utilized to examine differences across seasons and by sex for each segment.

## Results

Table 1 highlights the means and standard deviations across each time segment and season by sex. Paired samples *t* tests indicated that youth were significantly more active (p<0.05) during the Spring across all time segments except outside of school (p=0.33) and lunch recess (p=.18). Boys were more active during every segment except Fall PE (p=0.05), and Fall weekend (p=.47).

## Discussion

### In School and Out of School

Children spend approximately the same number of waking hours at school as they do out of school, yet, they accumulate greater steps outside of school. In the current study youth accumulated approximately 40% of their physical activity at school in the Fall compared to 47% in the Spring. This highlights the potential for additional physical activity programming while at school (Kulinna et al., 2012), especially when the weather is more extreme. The difference between in and out of school step counts narrowed when the weather become more suitable for physical activity in the spring. The weather in early Fall was in the 90’s F (average high temperature) and Spring weather was cooler with an average high temperature in the 70’s F. The cooler temperatures allowed for greater step counts during outdoor recess as well physical education classes being held outdoors which showed to increase youth physical activity (McKenzie et al., 1995). When compared to other school studies (Brusseau and Hannon, 2013) exploring in-school physical activity, it appears that children were in the typical US range during the Fall data collection and at the top or above the range (girls) during the Spring and both boys and girls were more in line with international school studies during the Spring. Seasonal findings (Beighle et al., 2008, 2012; Brusseau et al., 2012) are supported in this study illustrating that physical activity increases when the temperatures are more moderate (not too hot and not too cold). In school and out of school contributions in the Fall were consistent with previous research, yet in the Spring in school contributions were above the typical percentages (Beighle et al., 2012; Brusseau et al., 2011, 2013; Tudor-Locke et al., 2006). Findings in the current study were consistent with previous research with boys being generally more active both in school and outside of school (Tudor-Locke et al., 2009).

### Lunchtime and Afternoon Recess

Recess is an important segment for youth to be physically active (Erwin et al., 2012). Compared to previous work (Tudor-Locke et al., 2009), students accumulated a similar number of steps during lunch and afternoon recess.

Furthermore, lunch recess and afternoon recess contributed 13% and 10% of daily steps counts, respectively.

Previous research (Ridgers et al., 2006) has suggested that recess physical activity stays consistent across seasons. In our sample, lunch recess steps counts stayed consistent, however, the afternoon 15 min recess was significantly greater during the more mild Spring weather. Unfortunately, more and more schools are eliminating recess opportunities in favor of academic subjects. Recent research (Erwin et al., 2014; Escalante et al., 2014; Larson et al., 2014) suggests that recess interventions and programming can increase physical activity during this time segment highlighting that this existing segment has potential for increased activity. Boys were more active than girls which has been highlighted in numerous studies exploring recess and free play physical activity (Brusseau et al., 2013).

### Weekday and Weekends

Similar to previous findings, children accumulated significantly lower step counts on weekends (i.e. Brusseau et al., 2011; 2013; Comte et al., 2013; Duncan et al., 2006). When compared to international youth, the current sample fell well short of the daily averages of children in Sweden (Raustorpet al., 2004), New Zealand (Duncan et al., 2006), and Cyprus (Loucaides et al., 2004) illustrating the overall lag of physical activity in the US compared to other developed countries. This highlights the need for greater programming on these days as children appear not to make up for the typical physical activity they might accumulate during school. In the United States most school facilities are closed and locked on the weekends making use of these facilities for physical activity during this time difficult. Furthermore, these low activity levels may highlight the lack of facilities or social opportunities for play and an increase in parental safety concerns (Ding et al., 2011) when school access is not available.

### Physical Education (PE)

PE days were the greatest activity days which is consistent with previous work (Brusseau et al., 2011; 2013). PE also contributed approximately 16% of their daily step counts. Sex differences were non-existent during the Fall when PE was often taught in a more controlled gym environment. When PE was outside in the Spring, boys regained the physical activity advantage. It is important that PE offers equal physical activity opportunities for both boys and girls. PE was outdoor in the Spring which led to an increase of approximately 800 steps highlighting previous research (McKenzie et al., 1995) that youth are more active during outdoor PE classes. Previous research (Scruggs et al., 2003) would suggest that based on the steps/minute of PE youth were active (MVPA) less than 50% of class in the Fall and well over 50% in the Spring.

### Classroom Physical Activity

Very little physical activity came from in the classroom in this sample. Studies have begun to highlights the benefits of classroom activity breaks (Bershwinger and Brusseau, 2013; Goh et al., 2014) and physical activity and academic integration on daily physical activity patterns. Berschwinger and Brusseau (2013) found that classroom based physical activity had the ability to contribute an additional 1000 steps/day. Classroom physical activity has also been recommended as an important part of Comprehensive School Physical Activity Programming (Burns et al., 2015).

### Daily Recommendations

Previous research has highlighted a substantial disparity between actual physical activity patterns of youth and daily recommendations (Brusseau, Tudor-Locke, and Kulinna, 2013). The current sample met daily recommendations during weekday’s 46% Fall/59% Spring, weekend’s 12% Fall/21% Spring, 7 day week 36% Fall/47% Spring, and PE days 59% Fall and Spring. The current sample was falling well short of meeting the 12000 steps/day.

### Limitations

There are a few limitations that need to be noted. First, the study consisted of only two schools in one region of the US. This study would need to be replicated in other locations in the US and around the world for greater transferability. The study also used a spring levered pedometer which may underestimate physical activity in overweight individuals and does not measure MVPA or activities like cycling and swimming.

## Conclusions

This study provides important insight into the intricacies of children’s physical activity and highlights the potential seasonal impact across various activity segments. There is a clear need for Comprehensive School Physical Activity Programming and weekend physical activity opportunities. Furthermore, greater emphasis is needed on physical education and across other activity segments for girls to increase their physical activity levels.

## Figures and Tables

**Table 1:** Youth step counts during two seasons by sex

Fall	Physical Education	Lunch Recess	Additional Recess	In School	Out School	PE Weekdays	Weekdays	Weekends	Total Week
Boys	1645 (641)	1836* (525)	1164* (387)	5161* (1416)	7807* (3150)	14669* (5888)	12606* (3837)	8622 (6026)	11920* (3959)
Girls	1558 (603)	1351 (408)	946 (330)	4153 (1105)	6458 (2889)	12070 (4584	10291 (3343)	7715 (4604)	9780 (3294)
Total	1600 (623)	1555 (519	1038 (370)	4568 (1336)	6989 (3060)	13055 (5256)	11224 (3722)*	8033 (5164)	10647 (3723)
Spring	Physical Education^	Lunch Recess	Additional Recess^	In School^	Out School	PE Weekdays^	Weekdays^	Weekends^	Total Week^
Boys	2805* (1307)	1728* (569)	1386* (543)	6568* (2272)	7655* (3041)	15537* (5590)	14399* (4672)	8144* (4697)	13371* (4504)
Girls	2191 (1134)	1451 (472)	1310 (517)	6044 (1907)	6841 (3310)	13711 (4957)	12852 (4385)	7011 (3761)	11434 (3920)
Total	2379 (1219)	1563 (530)	1340 (527)	6253 (2072)	7136 (3231)	14226 (5185)	13411 (4542)	7346 (4073)	12131 (4233)

* significant difference by sex (p<0.05)

^ significant difference by season (p<0.05)
